# Effective recruitment strategies in an exercise trial for patients with fibromyalgia

**DOI:** 10.1186/s13063-021-05502-3

**Published:** 2021-08-21

**Authors:** Michelle Park, Raveendhara R. Bannuru, Lori Lyn Price, William F. Harvey, Jeffrey B. Driban, Chenchen Wang

**Affiliations:** 1 Tufts University School of Medicine, Boston, MA USA; 2 Center for Complementary and Integrative Medicine, Division of Rheumatology, Tufts Medical Center, Tufts University School of Medicine, 800 Washington Street, Box 406, Boston, MA 02111 USA; 3 Center for Treatment Comparison and Integrative Analysis, Division of Rheumatology, Tufts Medical Center, Boston, MA USA; 4 Tufts Clinical and Translational Science Institute, Tufts University, Boston, MA USA; 5 Institute for Clinical Research and Health Policy Studies, Tufts Medical Center, Boston, MA USA

**Keywords:** Fibromyalgia, Recruitment, Chronic pain, Pain management, Complementary and integrative health, Exercise trial

## Abstract

**Background:**

Recruitment of fibromyalgia populations into long-term clinical trials involving exercise interventions is a challenge. We evaluated the cost and randomization yields of various recruitment methods used for a fibromyalgia trial in an urban setting. We also investigated differences in participant characteristics and exercise intervention adherence based on recruitment source.

**Methods:**

We recruited individuals with fibromyalgia in the greater Boston area to a randomized controlled trial (RCT) using six recruitment strategies: newspaper advertisements, web advertisements, flyers, clinic referrals, direct mailing to patients in a clinic database, and word of mouth. We used the American College of Rheumatology 1990 and 2010 diagnostic criteria to screen and enroll participants. During an initial phone call to an interested participant, the study staff asked how they heard about the study. In this study, we compared the cost and yield of the six recruitment strategies as well as baseline characteristics, adherence, and attendance rates of participants across strategies.

**Results:**

Our recruitment resulted in 651 prescreens, 272 screening visits, and 226 randomized participants. Advertisements in a local commuter newspaper were most effective, providing 113 of 226 randomizations, albeit high cost ($212 per randomized participant). Low-cost recruitment strategies included clinical referrals and web advertisements, but they only provided 32 and 16 randomizations. Community-based strategies including advertisement and flyers recruited a more racially diverse participant sample than clinic referrals and mailing or calling patients. There was no evidence of difference in adherence among participants recruited from various strategies.

**Conclusions:**

Newspaper advertisement was the most effective and most expensive method per randomized participant for recruiting large numbers of individuals with fibromyalgia in an urban setting. Community-based strategies recruited a more racially diverse cohort than clinic-based strategies.

**Trial registration:**

ClinicalTrials.gov NCT01420640. Registered on 19 August 2011.

## Background

It is essential to use recruitment strategies that will yield a large and diverse group of eligible participants at minimal cost to conduct a clinical trial with results that are generalizable to the broader population. Effective strategies resulting in high recruitment can lead to shorter trial periods, thereby lowering the overall operational cost of the study [1].

A common recruitment strategy used in randomized controlled trials (RCTs) is direct invitational mailings to patients within an existing clinical practice [2–4]. Other recruitment methods rely on clinicians’ referral of patients to relevant clinical trials as well as print and online advertisements [5]. However, these strategies may not yield adequate numbers of participants for trials in certain populations, such as individuals with fibromyalgia. This population has historically been difficult to recruit into and retain in trials of physical activity [2, 6]. Exercise can result in an immediate worsening of pain and fatigue in fibromyalgia patients before improving their symptoms, challenging their tolerance of an exercise program [7]. The burden of pain, fatigue, stress, and depression often associated with the condition contribute to sedentary lifestyles in many patients [8, 9]. Moreover, these patients often have sporadic interactions with the healthcare system. Most patients see a rheumatologist for consultation but are routinely followed by primary care practitioners [10]. Many patients also seek care from various other healthcare providers (e.g., physical therapists, massage therapists, and complementary and integrative medicine care providers). This makes it challenging to recruit a large sample size from a specific clinical setting. Hence, this population is often more accessible through community-based recruitment than clinic-based recruitment [10]. Despite these challenges, effective recruitment strategies resulting in successfully completed clinical trials are vital to patients with fibromyalgia, as this population urgently needs effective treatment approaches to improve quality of life and reduce disability.

In this study, we report our successful recruitment strategies from a comparative effectiveness RCT examining the effects of Tai Chi and aerobic exercise for fibromyalgia [11, 12]. We evaluate the randomization yields and associated costs of multiple recruitment strategies. We also describe the baseline characteristics and adherence rates of participants recruited using these strategies.

## Methods

### Study design

The parent study investigating the comparative effectiveness of Tai Chi and aerobic exercise for fibromyalgia was conducted at Tufts Medical Center in Boston, Massachusetts. The rationale, design, and results of the Tai Chi and aerobic exercise for fibromyalgia study have been previously published [11, 12]. We enrolled 226 participants from March 2012 to August 2014, which exceeded the study’s enrollment goal of 216 participants. The RCT was divided into six enrollment cycles, with each cycle randomizing eligible participants into one aerobic exercise group and two of the four types of Tai Chi groups. For each of the six cycles, 50–60 individuals were scheduled to attend an initial baseline screening visit, and approximately 36 of those participants were eventually randomized into the study. Participants were enrolled into a 12-week (once or twice per week) or 24-week (once or twice per week) Tai Chi intervention or a 24-week twice per week aerobic exercise intervention, with follow-up at 52 weeks. Participants were evaluated before randomization at baseline, prior to beginning the study, and at 12 weeks, 24 weeks, and 52 weeks. This study was approved by the Tufts Health Sciences Institutional Review Board (approval #9945).

### Eligibility criteria

The eligibility criteria were designed to identify individuals with fibromyalgia who are 21 years or older and (a) fulfill the American College of Rheumatology (ACR) 1990 classification criteria for fibromyalgia [13] and (b) fulfill the ACR 2010 preliminary diagnostic criteria for fibromyalgia [14]. They were willing to complete the study and abstain from aerobic exercise or any other formalized exercise programs if assigned to Tai Chi and, conversely, abstain from Tai Chi if assigned to aerobic exercise. Participants were excluded if they had significant prior experience with Tai Chi or other types of complementary and integrative therapies in the past year and/or if they were diagnosed with any chronic or serious medical conditions known to contribute to fibromyalgia symptomatology or would limit the ability to participate in the Tai Chi or aerobic exercise programs.

### Recruitment strategies

We used a wide range of recruitment methods which we have categorized as clinic-based strategies and community-based strategies. Clinic-based recruitment strategies included referrals from clinicians both from our home institution and from the greater Boston area, and direct phone calls and mailings to patients from a patient database. Community-based recruitment strategies included web and print advertisements, flyers, and word of mouth referral. All mailings, advertisements, and flyers reported identical information describing inclusion criteria, estimated length of evaluation visits, and study contact information. During an initial prescreening phone call with each interested potential participant, the study staff asked how they heard about the study.

### Clinic-based recruitment

Internal referrals were received from three physicians supported by the NIH-funded study who were from the Tufts Medical Center rheumatology clinic, which serves approximately 500 fibromyalgia patients annually. All rheumatology clinicians at Tufts Medical Center were informed of the study and encouraged to mention it to eligible patients. The clinicians were also reminded of the study requirements in an email 1 month prior to the beginning of each recruitment cycle. External referrals were collected from two practicing rheumatologists in the Boston area who volunteered their time. Direct mailings were sent to patients selected from a 2500 patient database provided by one of the rheumatologists. The second rheumatologist provided a call list of 66 fibromyalgia patients who had previously consented to be contacted for future research studies. Given the on-site requirements of the aerobic exercise and Tai Chi classes during the study, mailings were sent to addresses with zip codes within a 20-mile radius of Tufts Medical Center. One thousand eighty of the 2500 patients had addresses listed within this radius. Selected patients were mailed a letter that briefly described the study and asked the patient to call the study phone line if they were interested in participating.

### Community-based recruitment

Standard quarter-page print advertisements were placed in a local newspaper *Metro Boston*. *Metro Boston* is a daily, free newspaper designed for a 20-min read for commuters. In the Boston area, *Metro Boston* receives 144,914 daily print and online readers, and adults aged 18–49 years old make up 57% of *Metro Boston's* readership [15]. This readership matched the target study population. A standard quarter-page advertisement was placed every working day for 4 weeks.

Standardized web advertisements were placed on Craigslist, an American website for classified advertisements, six times throughout the course of the study (once per recruitment cycle). The postings were made under the “volunteer” section in Craigslist. Similar web advertisements were also posted on Facebook, the Tufts Medical Center website, and *clinicalconnection.com* and *clinicaltrials.gov* throughout the duration of the trial.

Flyers were posted throughout Tufts Medical Center, Tufts University School of Medicine, Tufts University School of Dentistry, the local YMCA, and various community spaces, all within a half-mile radius of the hospital. The flyers were printed on brightly colored 8.5 × 11-inch paper.

Interested individuals also contacted study staff directly through word of mouth referrals from patients.

### Screening and randomization

Participants were prescreened over the telephone by a qualified and trained Study Coordinator, focusing on eligibility criteria that could be self-reported, including the presence of generalized body pain, fatigue, other diagnosed sources of chronic pain, and experience practicing mind-body therapies such as Tai Chi or yoga on a regular basis. Demographic information and source of recruitment were also collected.

Individuals who were eligible after the prescreening were invited to attend a screening visit, during which the Principal Investigator or Study Coordinator obtained written informed consent from the participant. Consented participants were assessed using the ACR 2010 classification criteria for fibromyalgia. If they met these criteria, participants were evaluated by the Study Physician based on the ACR 1990 criteria for fibromyalgia. If both 2010 and 1990 ACR criteria for fibromyalgia were met, the participant was provided a series of screening questionnaires and assessed by study nurses for physical function measurements and vital signs. Following the screening process, participants were randomized into intervention groups.

### Tracking costs

Recruitment costs were calculated by adding personnel, advertisement placement, paper, and postage costs. The costs for each recruitment method are detailed below.

#### Recruitment strategies

##### Patient database—phone calls

We estimated it took 1 h for initial calls to the 66 patients in the patient call database at $20/h of personnel cost. For some patients who did not answer the phone or whose phone number had changed, no further attempts were made.

##### Patient database—direct mailings

Personnel time for printing, sealing, and mailing letters was approximately 9 h per cycle at $20/h of personnel cost. Thus, the total personnel cost for mailing was estimated to be $1080. Postage was estimated as $540 at $0.50/letter for 1080 letters over the six cycles. An additional $120 was estimated for address labels and envelopes.

##### Web advertisements

Each posted advertisement on Craigslist incurred a cost of $25. One advertisement was placed on Craigslist per study recruitment cycle, for a total of six advertisements posted over the course of the study ($150). The study was additionally advertised on Facebook, the Tufts Medical Center website, *clinicaltrials.gov*, and *clinicalconnection.com* at no cost. Personnel costs were negligible for web advertisements compared to posting flyers and newspaper advertisements, as most of the materials were recycled.

##### Newspaper advertisements

Twenty newspaper advertisements were placed each cycle for six cycles at $200/advertisement (120 advertisements in total for $24,000).

##### Flyers

Personnel time and costs for posting flyers were estimated as 1 h/week ($20/h) for 8 weeks prior to the beginning of each of the six study cycles ($960). Paper cost was $4.00/ream of paper (500 sheets), with an estimated use of 1 paper packet/study cycle ($24).

##### Clinical referral and word of mouth recruiting

These strategies incurred no costs.

### Statistical analyses

The effectiveness of each recruitment method was assessed by calculating randomization yield, which was defined as the percentage of participants eventually randomized out of those prescreened. We calculated yield out of those prescreened because the number of individuals who call to inquire about the trial and complete a prescreen reflects the most immediate result of our recruitment efforts. During this initial phone call with an interested participant, the study staff asked how they heard about the study. Only one recruitment strategy was attributed to each participant. We assessed the total cost and cost per randomization for each recruitment strategy. Descriptive statistics were used to outline the characteristics of participants recruited by each method. Participant characteristics provided include age, gender, race, number of years since diagnosis, body mass index, and education level. We also calculated adherence rate, defined as the mean percentage of classes attended out of the total offered. The percentage of participants who attended each outcome evaluation at 12, 24, and 52 weeks is also reported.

## Results

**Fig. 1:**
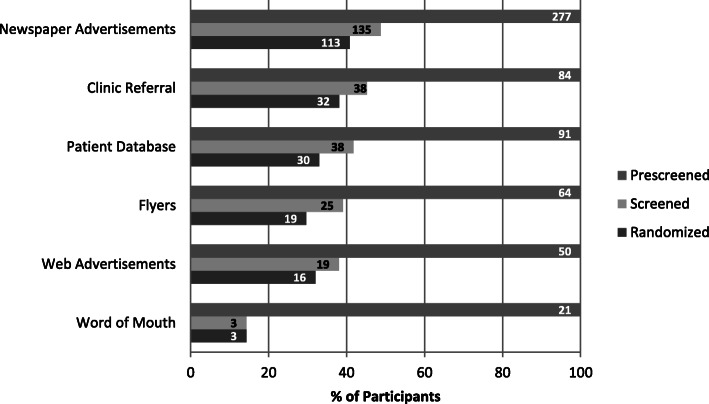
Yields per recruitment strategy. Numbers in bars indicate absolute numbers of participants prescreened, screened, and randomized through each strategy. Lengths of bars represent the percentage of participants who were screened or randomized out of the total prescreened (100%) by each strategy

**Fig. 2:**
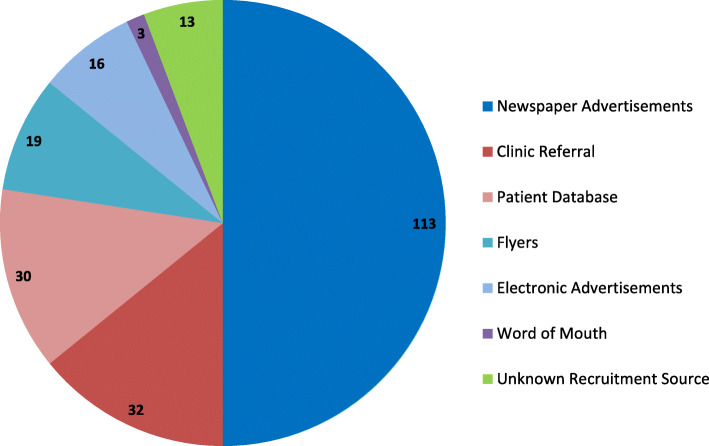
Randomized participants by recruitment strategy. Number of randomized participants yielded from each recruitment strategy (total *n* = 226)

In total, 651 participants were prescreened and 272 participants were screened over the 30-month recruitment period of the study. A total of 226 participants were randomized by August 2014. The numbers of prescreened, screened, and randomized participants derived from each recruitment method are summarized in Fig. 1. There was no information about the recruitment method for 64 participants who were prescreened (9.8% total prescreened), 14 participants who attended a screening visit (5.2% total attended screening visit), and 13 participants who were randomized into the study (5.8% total randomized to study). Figure 2 illustrates the numbers of participants randomized from each recruitment strategy. Newspaper advertisements resulted in the most randomizations at 113 participants, which accounted for half of all randomized participants. Clinical referrals and direct mailings resulted in 32 and 30 randomizations, respectively. The remaining strategies each resulted in 19 randomizations or less. Randomization yield (percentage randomized out of total prescreened) was similar across all recruitment strategies, ranging from 30 to 40% with the exception of word of mouth referral which had a lower yield (Table 1). Thus, the differing levels of randomization among recruitment strategies demonstrated in Fig. 2 were driven by the initial reach of the strategy to reach patients as opposed to yield from prescreen onward.

**Table 1:** Randomization yields and costs of recruitment strategies

Recruitment strategy	Number of randomized participants	Randomization yield *n*/*m* (%)	Total cost	Cost per randomization
Newspaper advertisement	113	113/277 (40.8%)	$24,000	$212.39
Clinical referral	32	32/84 (38.1%)	$0	$0
Patient database	30	30/91 (33.7%)	$1740	$58.00
Flyers	19	19/64 (29.7%)	$984	$51.79
Web advertisement	16	16/50 (32%)	$150	$9.38
Word of mouth	3	3/21 (14.3%)	$0	$0
Unknown	13	13/64 (20.3%)	N/A	N/A
**All strategies**	226	226/651 (34.7%)	$26,874	$118.91

*n*, randomized participants; *m*, prescreened participants; *%*, percent randomized out of total prescreened

Total cost based on personnel, advertisement, and material costs for all recruitment methods was $26,874 (Table 1). Consequently, the average recruitment cost per randomized participant was $118.91. Newspaper advertisements incurred the highest cost ($212/participant) but also produced 50% of all randomizations. Although clinical referrals and patient database communications yielded similar numbers of randomizations (32 vs. 30 participants), patient database communications were more expensive at $58/randomization as opposed to clinical referrals which had no associated cost. Flyers and web advertisements also yielded similar numbers of randomizations (19 vs. 16 participants), but web advertisements were less costly ($9 vs. $52).

**Fig. 3:**
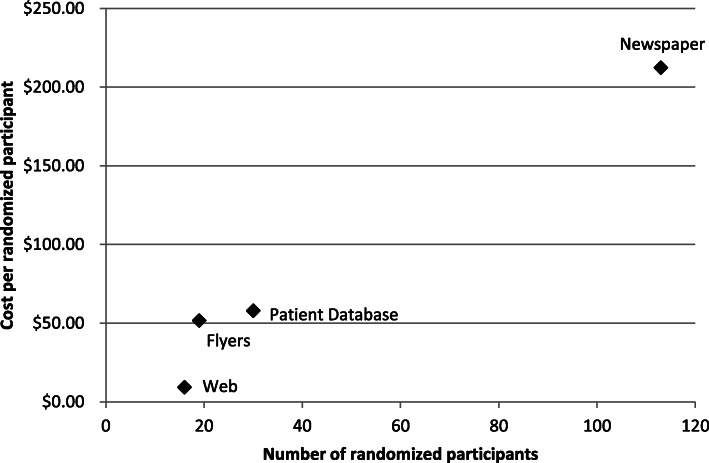
Relationship between cost and number of randomizations for recruitment strategies. Note: Strategies not shown here, clinical referrals and word of mouth referrals, incurred $0 in recruitment costs

Of the four strategies with controllable costs, strategies in order of increasing cost per randomization were web advertisement, flyers, patient database communication, and newspaper advertisement (Table 1). Of note, however, numbers of randomizations from the strategies also increased with increasing cost (Fig. 3). For example, although web advertisements recruited one randomized participant for every $8 incurred, it only resulted in 18 randomizations total. On the other hand, newspaper advertisements yielded one randomized participant for every $212, but the strategy amassed 113 participants in total. Thus, low-cost strategies were less effective, whereas newspaper advertisements were high-cost but considerably more successful.

All strategies recruited participants that were similar in age, gender, body mass index, and educational background (Table 2). Participants across recruitment strategies reported similar average numbers of years since fibromyalgia diagnosis except for those from patient databases, who tended to have longer average durations since diagnosis (11.4 years). Notably, advertisement in the community recruited a more racially diverse group of participants (50 to 54% white participants from newspaper and web advertisements, and flyers) than clinic-based strategies (69% white participants from clinical referrals and 93% from patient database).

**Table 2:** Characteristics of randomized participants by recruitment strategy^a^

Recruitment strategy	Age, years	Female (%)	Race (%)			Duration of pain, years	Body mass index, kg/m^2^	Education level (%)		
			White	Black	Others			High school	College	Graduate school
Newspaper advertisement (*n* = 113)^b^	52.2 ± 11.9	92.0	54.0	28.3	17.7	8.9 ± 7.1	29.8 ± 6.4	22.1	61.1	16.8
Clinical referral (*n* = 32)	50.5 ± 12.8	100.0	68.8	21.9	6.3	7.4 ± 7.8	31.9 ± 6.7	25.0	59.4	12.5
Patient database (*n* = 30)	53.7 ± 12.0	93.2	93.3	6.7	0.0	11.4 ± 8.0	30.8 ± 8.2	13.3	56.7	30.0
Flyer (*n* = 19)	49.7 ± 13.0	79.0	52.6	15.8	31.6	7.3 ± 6.6	28.8 ± 5.2	21.1	63.2	15.8
Web advertisement (*n* = 16)	47.7 ± 14.0	93.8	50.0	25.0	25.0	7.8 ± 10.5	27.2 ± 5.9	6.7	60.0	33.3
Word of mouth (*n* = 3)	45.0 ± 6.0	100.0	33.3	33.3	33.3	10.3 ± 0.6	30.9 ± 6.6	33.3	33.3	33.3
Unknown (*n* = 13)	54.2 ± 11.0	92.3	61.5	23.1	15.4	7.1 ± 6.1	31.0 ± 8.3	0.0	69.2	30.8

^a^Values are reported as mean ± SD unless otherwise noted

^b^*n*, number of randomized participants from recruitment strategy

Overall, all recruitment strategies yielded similarly adherent cohorts in terms of exercise class attendance (Table 3). Web advertisements recruited the most adherent participants, as they attended an average of 66% of classes. Participants recruited through word of mouth and clinical referrals were the least adherent, having attended an average of 49 and 51% of classes. No notable differences were observed among recruitment strategies in outcome evaluation attendance at 12, 24, or 52 weeks.

**Table 3:** Adherence and outcome evaluation attendance by recruitment strategy

Recruitment strategy	Adherence rate^a^ (mean ± SD)	Never attended outcome evaluation^b^ (%)	Attendance rate (%)		
			12-week evaluation	24-week evaluation	52-week evaluation
Newspaper advertisement (*n* = 113)^c^	52.7 ± 32.9	8.0	81.4	77	68.1
Clinical referral (*n* = 32)	50.5 ± 32.1	6.3	78.1	78.1	65.6
Patient database (*n* = 30)	59.2 ± 29.7	6.7	76.7	80.0	73.3
Flyers (*n* = 19)	59.1 ± 23.8	5.3	84.2	84.2	84.2
Web advertisement (*n* = 16)	65.9 ± 27.2	12.5	75	81.3	56.3
Word of mouth (*n* = 3)	49.4 ± 31.0	0.0	100	100	66.7
Unknown (*n* = 13)	59.1 ± 28.4	7.7	92.3	92.3	84.6

^a^Treated participants only; adherence rate defined as mean percentage of classes attended out of total classes offered

^b^Participants who did not attend the 12-, 24-, or 52-week outcome evaluations

^c^*n*, number of randomized participants from recruitment strategy

## Discussion

The purpose of this study was to examine the strategies used to recruit participants into a comparative effectiveness RCT examining the effects of Tai Chi and aerobic exercise for fibromyalgia. The most effective strategy, newspaper advertisements, was also the most expensive. It yielded half of the total randomizations, costing $212/participant. The least effective strategy, word of mouth recruitment, was also among the lowest in cost. There was no notable difference in randomization yield among recruitment strategies. While clinic-based strategies were less expensive overall, advertisement in the community drew in more participants and yielded more racial diversity.

In general, higher recruitment numbers were associated with higher cost recruitment strategies. As noted above, the most successful, albeit most expensive, recruitment strategy was advertising in a local commuter newspaper, which recruited half of the participants randomized into the study (*n* = 113). Clinical and word of mouth referrals were the least costly recruitment strategy as they had no associated costs. However, clinical referrals recruited only about a quarter of the participants (*n* = 32) that newspaper advertisements did. Furthermore, word of mouth referral had the lowest retention of participants from prescreening (14% randomization yield), perhaps due to the potential lack of information or misinformation that can occur through word of mouth referral. Mailings and phone calls to patients from a patient database recruited a similar number of participants as clinical referrals but were more expensive. Additionally, mailings tended to generate a high volume of prescreens mainly in the first 2 to 3 weeks after mailing, while clinical referrals provided a small, steady influx of participants. Web advertisements, despite being placed only eight times throughout the trial, and flyers, which were posted weekly, were both moderately useful strategies that were lower cost per randomized participant than newspaper advertisements or patient database mailings and calls. To maximize recruitment, future studies may benefit from placing web advertisements more frequently. However, it should be noted that the web and social media landscapes have changed since the time of our recruitment. With the increased consumption of advertisements via social media platforms as opposed to websites such as Craigslist [16], the most appropriate platforms for online advertisement in future trials may vary based on when recruitment occurs as well as the target population.

All strategies recruited participants that were similar in terms of age, gender, years since diagnosis, body mass index, and education level; however, community-based strategies including advertisements and flyers recruited a more racially diverse population that was representative of the greater Boston area demographics of 2010 [17]. Compared to fibromyalgia patients nationally, the randomized population in our RCT as a whole was similar in age, gender, BMI, and education, but more racially diverse [18, 19]. The ratio of women to men prescreened, screened, and randomized into the study was approximately 9:1, consistent with estimates of fibromyalgia prevalence by sex in the USA which ranges from 7:1 to 9:1 [20]. In general, we found that participants from various recruitment methods were similar in demographic characteristics, except for race. Adherence, defined as attendance of classes, and attendance at outcome evaluations were also similar among participants recruited through different methods. Thus, recruitment methods may influence the racial makeup of a study sample but likely do not affect the adherence of participants who are recruited.

One previous fibromyalgia study that described their recruitment strategy focused entirely on maximizing the recruitment yield following mailing invitations to patients from a database [2]. Their strategy of direct mail recruitment from a patient database yielded a 7% randomization of all contacted individuals, whereas the same strategy for us yielded a 1% randomization. In comparison, we used multiple recruitment strategies of which direct mailings only accounted for 28 of 226 randomizations. Another recruitment study including two integrative therapy RCTs in chronic low back pain patients reported a lower percentage of their randomized participants recruited by newspaper advertisement than in our RCT. The two RCTs in the study obtained 57% of randomizations from direct mailing recruitment at a cost of $325 per randomized participant [5]. Twenty-eight percent of randomizations came from newspaper advertisements at a cost of $400 per randomization. In contrast, 12% of our randomizations came from direct mailings at approximately $60 per randomization, whereas newspaper advertisement accounted for 50% of randomized participants at $212 per randomization. Recruitment for the low back pain RCTs occurred in 2004, about a decade earlier than our RCT. Juxtaposed with our recruitment, the results suggest that while newspaper advertisement still remains more expensive than direct mailings, it may have evolved to be a more successful strategy for clinical trial recruitment in the past decade. Balancing the lower cost of direct mailings and the effectiveness of newspaper advertisement may be key to efficient recruitment in future studies. We also believe that for our RCT, the newspaper’s reach to the large commuter population in the greater Boston area contributed to its success in recruitment. The newspaper that posted our advertisements was the *Metro Boston*, a free newspaper distributed at transit stations which received 144,914 daily print and online readers, with adults aged 18–49 years old composing 57% of the readership [15]. This readership both matched the target study population and had access to the city of Boston where our trial was located. Our findings have implications for recruitment in other RCTs but may not be applicable to some cities in which the local newspapers or flyers have less reach.

Chronic widespread pain including fibromyalgia syndrome is present in 10 to 15% of the population from countries throughout the world [21]. RCTs studying patients with fibromyalgia share similar recruitment barriers to RCTs concerning patients with chronic widespread pain. Patients living with other chronic diseases causing fatigue, pain, and psychological stress are similarly difficult to recruit into trials of physical activity [5, 22, 23]. Our study may be helpful for planning clinical trials in these patient populations as well. Moreover, our study shows that community-based recruitment strategies were successful in fibromyalgia patients, which may relate to the fact that many fibromyalgia patients seek multimodal care with complementary and integrative medicine providers, physical therapists, and massage therapists in the community [10]. Thus, the recruitment strategies in this study may be useful for trials in other populations that utilize similar patient care pathways, such as patients with chronic widespread pain. However, it must be noted that fibromyalgia patients are unique in that many expect doubt regarding the veracity of their condition from the medical community, which is what prevents some in this population from seeking consistent conventional medical care. It follows that patients with fibromyalgia may find the treatment arm of Tai Chi, a mind-body exercise therapy, more appealing than other patients, and this may have impacted their recruitment to the trial.

A limitation of this study was that the pool of prescreened participants was constrained to participants who completed the prescreen form over the phone. In some instances, potential participants who called to inquire about the study were excluded without formally completing a prescreen interview, as exclusion criteria were fulfilled based on the conversation surrounding the inquiry. These individuals were not counted in the number of prescreened participants. Given randomization yield is calculated as *n*/*m* × 100, in which *n* = number randomized and *m* = number prescreened, the denominator (*m*) would have been higher had we accounted for all those who started the prescreening process. In turn, the randomization yields would have been lower than currently reported. Also, some participants included in the analysis had incomplete data on recruitment source (*n* = 13), which may have obscured additional relevant information for our study. In addition, some recruitment strategies such as word of mouth only resulted in a few randomized participants, limiting the meaningfulness of values such as randomization yield and cost per randomization of those strategies. Finally, the strategy of advertisements in a commuter newspaper may not be as effective in suburban or rural areas or in areas with a small commuter population. It must be noted that the effectiveness of newspaper advertisements and flyers is likely variable depending on location. Although our recruitment was conducted in an urban area, similar strategies may still be effective in rural and suburban areas. However, success will depend on the familiarity of study staff with how their community engages with print and digital media, and recruitment in these areas warrants further study. The US health context may also impact the effectiveness of recruitment strategies. Our strategies were effective for a commuter population in the greater Boston area but may not apply globally. However, the two-pronged approach of community-based and clinic-based recruitment potentially overcame some of the barriers to participation due to the lack of access to healthcare institutions in the USA.

Despite these limitations, there were several strengths of this study, including the large size of the cohort. As one of the largest exercise RCTs to date in a fibromyalgia population, this study’s recruitment is important to study to inform the conduct of future large RCTs for fibromyalgia. Furthermore, as fibromyalgia is increasingly recognized as a condition necessitating non-pharmacological therapeutic approaches, understanding how to successfully recruit this patient population into exercise RCTs will be essential for future research [24]. Another strength of this study was the continual, detailed tracking and reporting of recruitment source information. Detailed tracking allowed us to critically examine the factors that may influence recruitment in this traditionally difficult-to-recruit population.

## Conclusions

In summary, advertisement in a commuter newspaper was the most effective strategy per randomized participant to recruit fibromyalgia patients for an exercise RCT in an urban center. Future studies conducted in an urban location may find the most successful recruitment strategy includes targeted use of the more expensive but effective method of newspaper advertisement, combined with more frequent use of the lower cost and moderately effective strategies of flyers and web advertisements. The best platform for web advertisement may include more social media, consistent with the current landscape of media consumption. Established recruitment methods such as direct mailings and clinic referrals remain successful and should be used. For a study on a limited budget, efforts should be made to maximize clinical referrals and utilize frequent low-cost web advertisements. Patients with fibromyalgia urgently need effective treatment options, so continued effort to improve recruitment of this historically challenging-to-recruit population into exercise trials is vital. Effective recruitment strategies for trials in fibromyalgia patients would lead to shorter trial periods, thereby lowering the overall operational cost of the study and delivery of both pharmacological and non-pharmacological treatments to this population.

## Data Availability

The datasets used and/or analyzed during the current study are available from the corresponding author on reasonable request.
